# Supplementary material for: Functionalization of ZnAl-Layered Double Hydroxide with Ensulizole and Its Application as a UV-Protective Agent in a Transparent Polymer Coating
Source: Molecules. 2023 Aug 26;28(17):6262. doi: 10.3390/molecules28176262 (PMC10488424; doi:10.3390/molecules28176262)
Supplement: Supplementary file 1 [file molecules-28-06262-s001.zip › molecules-2558156-supplementary.pdf]

Functionalization of ZnAl-layered Double Hydroxide with Ensulizole and Its Application as UV-Protective Agent in a Transparent Polymer Coating

Klára Melánová [1], Kateřina Kopecká [2], Ludvík Beneš [1], Petr Kutálek [1], Petr Knotek [3], Zuzana Zmrhalová [1] and Jan Svoboda [4,*]

[1] Joint Laboratory of Solid State Chemistry, Faculty of Chemical Technology, University of Pardubice, Studentská 84, 53210 Pardubice, Czech Republic; klara.melanova@upce.cz (K.M.); ludvik.benes@upce.cz (L.B.); petr.kutalek@upce.cz (P.K.); zuzana.zmrhalova@upce.cz (Z.Z.)

[2] SYNPO, akciová společnost, S. K. Neumanna 1316, 53207 Pardubice, Czech Republic; katerina.kopecka@synpo.cz

[3] Department of General and Inorganic Chemistry, Faculty of Chemical Technology, University of Pardubice, Studentská 573, 53210 Pardubice, Czech Republic; petr.knotek@upce.cz

[4] Institute of Organic Chemistry and Technology, Faculty of Chemical Technology, University of Pardubice, Studentská 573, 53210 Pardubice, Czech Republic

* Correspondence: jan.svoboda@upce.cz; Tel.: +420-466-037-040

Supporting information

Table S1. Results of elemental analysis.

Sample	C%		H%		N%	
	found	calc	found	calc	found	calc
ZnAl–PBISA–1	23.50	23.38	3.57	3.75	4.15	4.12
ZnAl–PBISA–2	20.41	20.68	3.51	3.64	4.05	3.99
ZnAl–PBISA–3	25.02	24.37	3.44	3.75	4.28	4.31

Table S2. Found and calculated weight losses of the intercalates.

Sample	weight loss							
	first		second		third		total	
	found	calc	found	calc	found	calc	found	calc
ZnAl–PBISA–1	12.7	11.78	11.0	11.25	40.7	40.23	64.4	63.26
ZnAl–PBISA–2	12.1	12.62	11.6	12.83	36.6	35.12	60.3	60.57
ZnAl–PBISA–3	11.4	11.47	8.2	10.73	42.7	42.09	62.3	64.29

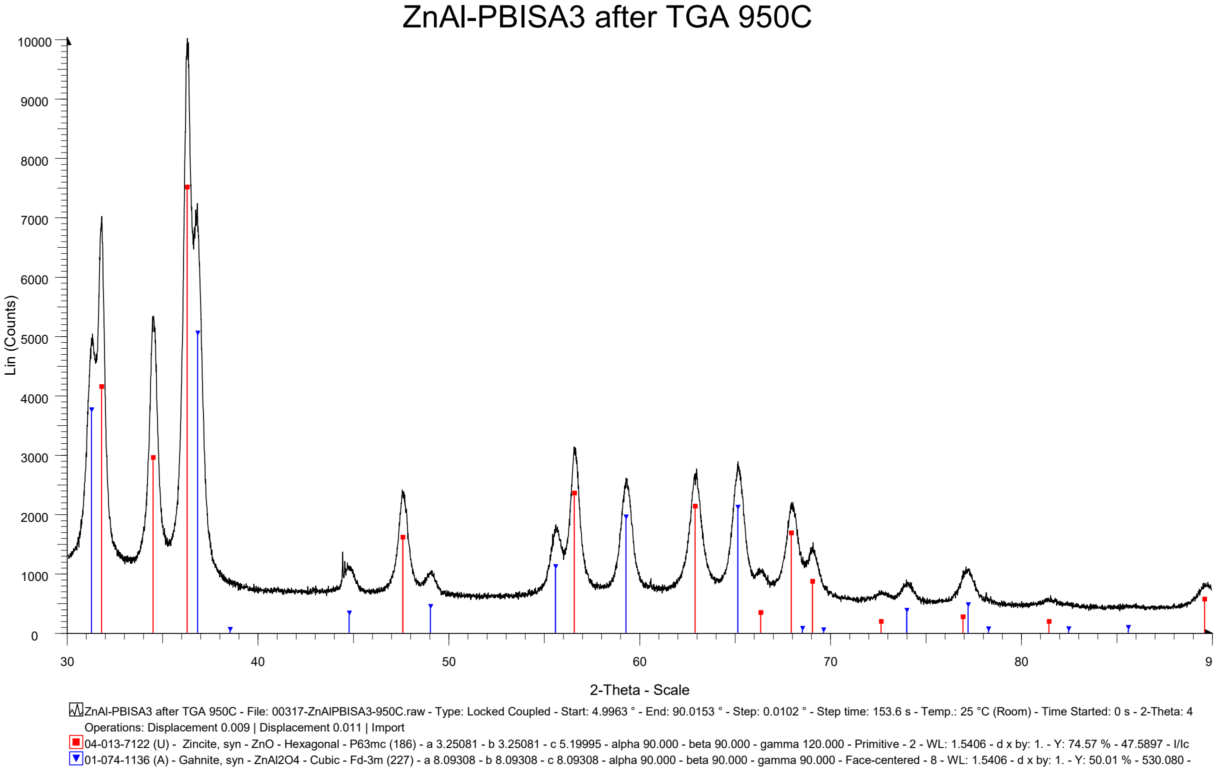

Figure S1. Powder X-ray diffraction pattern of the product of thermal deposition of ZnAl–PBISA–3.

The mass concentrations of the components in the solids after thermogravimetric analyses were calculated using the corundum method in the EVA program [1] by optimizing of the measured diffractogram. For each standard, the EVA program optimizes the scale factor and parameters describing the width of the diffraction lines, and the result is converted to concentrations using corundum numbers I/Icor from the PDF-4+ database [2]. During the calculation, the lattice parameters are also optimized. The optimization result is shown below:

Sample name: ZnAl–PBISA–3 after TGA 950C
File name: 00317-ZnAl–PBISA–3–-950C.raw
Date of fitting: 5.5.2023 12:17:50
Fitting limits: 29.5992 89.9431
Number of steps: 40
R/R0: 2.34
RWP: 6.93
Delta displacement: 0.009 mm

Concentrations:

04-013-7122	50.3%	ZnO Zincite; syn
01-074-1136	49.7%	ZnAl2O4 Gahnite; syn

O	27.2%		
Al	14.6%	Al2O3	27.6%
Zn	58.1%	ZnO	72.4%

04-013-7122 Zincite, syn
FWHM(30): 0.434°
Crystallite Size (Scherrer): 187.5 Å
I/Icor: 5.5
System: Hexagonal
Space group: P63mc (186)

Cell param.:	Initial	Final
a:	3.25087	3.25081
c:	5.20004	5.19995

01-074-1136 Gahnite, syn
FWHM(30): 0.488°
Crystallite Size (Scherrer): 166.7 Å
I/Icor: 3.8
System: Cubic
Space group: Fd-3m (227)

Cell param.:	Initial	Final
a:	8.09322	8.09308

After converting mass % to molar %, the Zn/Al ratio is 0.62/0.38, which is in good agreement with the Zn/Al ratio in the prepared intercalate.

1. EVA, ver.19. Diffrac plus Basic Evaluating Package, Bruker AXS GmbH, Germany, 2013.

2. Database PDF-4+, Joint Committee on Powder Diffraction Standards, International Centre of Diffraction Data, Swarthmore, PA.

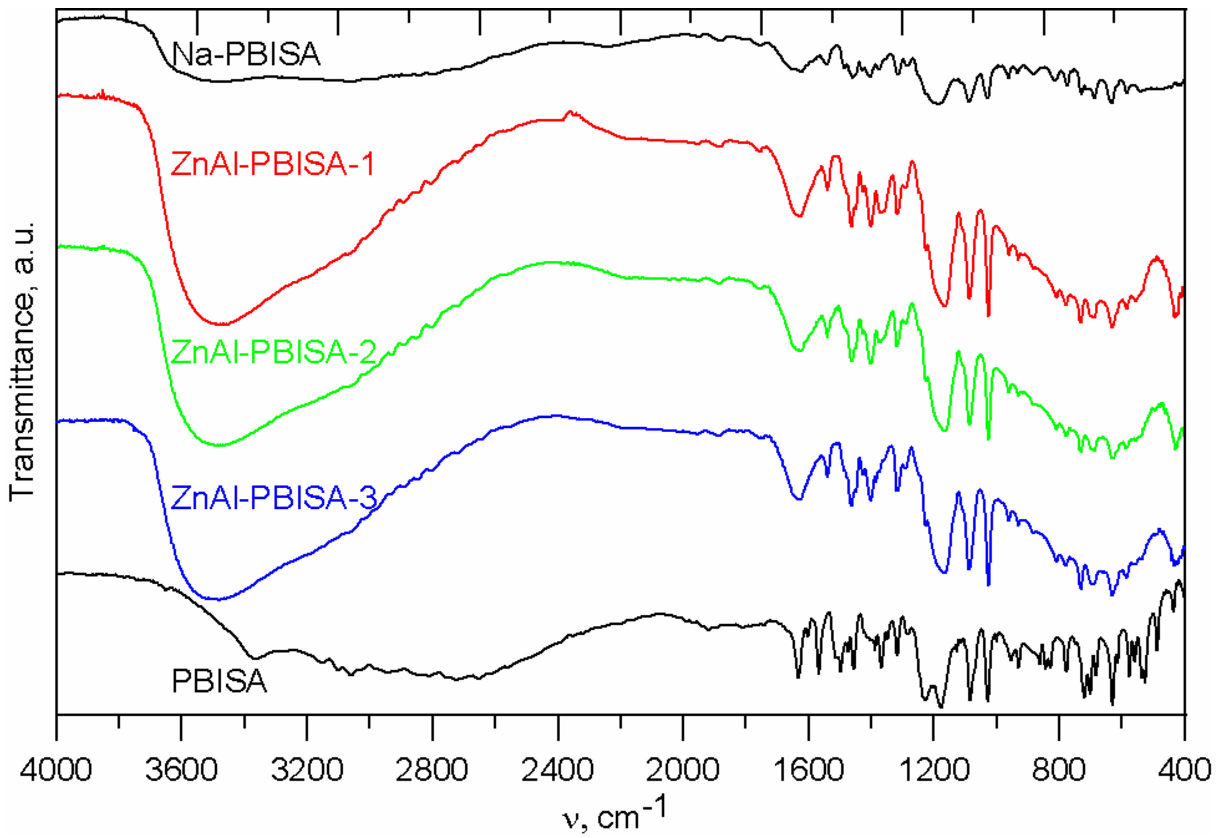

Figure S2. Infrared spectra of the intercalates in whole range.
